# Perioperative and Oncological Outcome of Laparoscopic Resection of Gastrointestinal Stromal Tumour (GIST) of the Stomach

**DOI:** 10.1155/2009/286138

**Published:** 2009-03-26

**Authors:** Ulrich Ronellenfitsch, Wilko Staiger, Georg Kähler, Philipp Ströbel, Matthias Schwarzbach, Peter Hohenberger

**Affiliations:** ^1^Division of Surgical Oncology and Thoracic Surgery, University Medicine Mannheim, Ruprecht-Karls-University Heidelberg, Theodor-Kutzer-Ufer 1-3, 68135 Mannheim, Germany; ^2^Department of Surgery, University Medicine Mannheim, Ruprecht-Karls-University Heidelberg, Theodor-Kutzer-Ufer 1-3, 68135 Mannheim, Germany; ^3^Institute of Pathology, University Medicine Mannheim, Ruprecht-Karls-University Heidelberg, Theodor-Kutzer-Ufer 1-3, 68135 Mannheim, Germany

## Abstract

*Background*. Surgery remains the only curative treatment for gastrointestinal stromal tumour (GIST). Resection needs to ensure tumour-free margins while lymphadenectomy is not required. Thus, partial gastric resection is the treatment of choice for small gastric GISTs. Evidence on whether performing resection laparoscopically compromises outcome is limited. *Methods*. We compiled patients undergoing laparoscopic resection of suspected gastric GIST between 2003 and 2007. Follow-up was performed to obtain information on tumour recurrence. *Results*. Laparoscopic resection with free margins was performed in 21/22 patients. Histology confirmed GIST in 17 cases, 4 tumours were benign neoplasms. Median operation time and postoperative stay for GIST patients were 130 (range 80–201) mins and 7 (range 5–95) days. Two patients experienced stapler line leakage necessitating surgical revision. After median follow-up of 18 (range 1–53) months, no recurrence occurred. *Conclusions*. Laparoscopic resection of gastric GISTs yields good perioperative outcomes. Oncologic outcome needs to be assessed with longer follow-up. For posterior lesions, special precaution is needed. Laparoscopic resection could become standard for circumscribed gastric GISTs if necessary precautions for oncological procedures are observed.

## 1. Introduction

Gastrointestinal stromal tumours (GISTs) are the most common mesenchymal neoplasms of the digestive tract with an estimated annual incidence of 10–20 cases per one million inhabitants [1, 2]. GISTs probably arise from precursor cells of the interstitial cells of Cajal. Their defining characteristic is a gain-of-function mutation in genes coding for the KIT tyrosine kinase receptor, which is considered the driving force of cell proliferation in this tumour [3]. Clinical presentation of GISTs ranges from indolent, hardly proliferating to fast-growing, recurring and metastasising tumours [1]. Fletcher et al. proposed a classification of aggressive behaviour for GISTs based on their maximum diameter and mitotic rate [4] (Table 1), factors which were both shown to predict recurrence and survival [5, 6].

Treatment of choice for primary GISTs remains complete resection. Whereas current National Comprehensive Cancer Network (NCCN) guidelines [7] recommend surgery for GISTs or supposed GISTs of any size, the latest European Society for Medical Oncology (ESMO) Clinical Recommendations stipulate frequent surveillance without surgery for lesions with a diameter below 2 cm [8]. In contrast to resection of intestinal carcinomas, surgery of GISTs does not require lymphadenectomy since lymphatic metastatic spread is extremely rare in primary tumours [1]. Thus, local resection of the tumour with clear margins is recommended. Moreover, strict avoidance of intraoperative tumour rupture is crucial for preventing tumour relapse.

About 50% of GISTs are located in the stomach which makes it the most frequent site of manifestation [2, 9]. Due to the often fragile consistence, particularly of pedunculated GISTs, there is an ongoing debate whether surgical resection of gastric GISTs can be performed laparoscopically without increasing perioperative morbidity and compromising oncological outcome. The latest ESMO Clinical Recommendations consider a laparoscopic approach “if cancer surgery principles are respected.” [8] Current NCCN guidelines do not contain a clear statement on whether surgery for GIST should be performed laparoscopically or through open surgery but recommend that surgery should produce minimal surgical morbidity [7].

The present study tries to evaluate whether laparoscopic resection of gastric GISTs can become a standard treatment for such tumours by analysing perioperative characteristics and long-term oncological outcome.

## 2. Material and Methods

### 2.1. Study Population and Data Analysis

The study includes all patients who underwent laparoscopic resection of a primary tumour of the stomach deemed to be a GIST on clinical assessment between January 1, 2003, when a laparoscopic approach became our standard for the described lesions, and December 31, 2007. Patients were eligible for laparoscopic surgery if preoperative staging (endoscopy, endosonography and CT scan) showed a localised, non-metastatic extramucosal gastric lesion. Tumours were required to be of a diameter and in a position which suggested resectability through segmental or wedge resection. Preoperative histological confirmation of the diagnosis was not a prerequisite for inclusion. Histological diagnosis of GIST was obtained from the resection specimen by means of hematoxylin and eosin staining and immunohistological assays for CD117 and CD34 and platelet-derived growth factor receptor alpha. Mutational analysis was performed where required.

From a prospectively kept database, we extracted the following characteristics: age, sex, length of postoperative hospital stay, operation time, tumour location, tumour size, classification of aggressive behaviour as defined in Table 1, histopathological assessment of resection margins, intraoperative blood loss, and incidence of perioperative complications (cardiac, pulmonary, septic, anastomotic failure, reoperation needed). We present single values as well as the median plus range or, where applicable, percentages, for the respective variables.

All patients with confirmed GIST were followed up regularly including upper GI endoscopy and abdominal CT scans every three to six months depending on the risk for malignant behaviour. From these visits we assessed vital status and tumour recurrence. A few patients with very low-risk GIST decided against adhering to this program and were followed up through phone calls to their general practitioner or themselves. In the former case, the family physician was asked when the patient had last presented and if to his knowledge any recurrence of the tumour was known. The same question was asked directly to patients in case their family physician was not available. Date of follow-up was ascertained either as the date of the patient's last visit to our outpatient clinic or the date of the phone call to the family physician or patient, respectively.

### 2.2. Surgical Methodology

**Figure 1:**
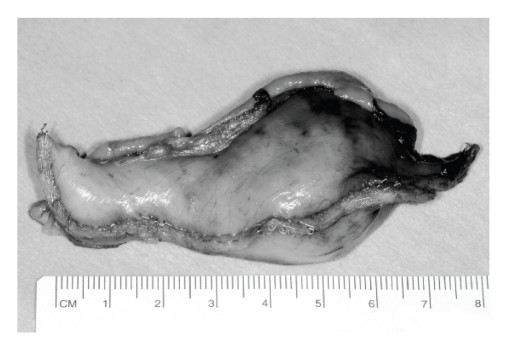
Endoscopic ink staining of tumour margins.

All laparoscopic resections were performed in a standardised manner. The patient was placed in supine position with legs spread and a four-port technique was used. After visual and tactile control of the liver and the abdominal cavity for metastases, the lesion was identified through visualisation and palpation. In case of tumour location at the posterior wall of the stomach, the gastrocolic ligament was dissected and the stomach inverted in order to display the lesion. If necessary, intraoperative endoscopy was performed to elevate the tumour and stain its margins with ink (Figure 1). The tumour-bearing gastric segment was resected with one or several 45 mm endoscopic linear staplers. The resection specimen was placed into a plastic retrieval bag and removed through one of the port incisions. Stapler lines were visually controlled and, if deemed necessary by the surgeon, their integrity was assessed through the application of methylene blue via a nasogastric tube. If the lines showed leakage or were deemed to be at risk for it, additional manual sewing of the line was performed. A postoperative control of stapler lines by means of endoscopy or CT scan after oral intake of contrast medium was only done in case of clinical suspicion of leakage.

## 3. Results

22 patients had been planned for laparoscopic segment resection of a suspected gastric GIST. Out of these, 21 were resected with the technique. In one patient, conversion to laparotomy was necessary due to extensive intraabdominal adhesions with the larger omentum completely fixed to the ventral abdominal wall. This patient was not included in the analysis. One of the 21 patients had received preoperative imatinib treatment for a GIST with a diameter of 8 cm. He had adverse characteristics for open surgery (pronounced obesity) and was reluctant to undergo any resection at first. Neoadjuvant imatinib caused a reduction of tumour size which enabled a laparoscopic approach to which the patient finally agreed. Histology confirmed a GIST in 17 of 21 cases. In one case histological workup showed pancreatic heterotopy, in one case leimoyoma, in one case gastric schwannoma, and in one case gastric wall lipoma.

Table 2 provides perioperative characteristics of the 17 GIST patients in which the intervention could be performed laparoscopically. Both sexes were equally represented and most patients were in their sixth or seventh decade of life (age range 43–79 years). Tumours were located in all parts of the stomach with a predominance of the corpus and antrum. All tumours were resected with negative margins and there was no intraoperative tumour rupture. Intraoperative blood loss was below 200 mL in all patients and no patient required blood transfusion. The median duration of surgery was 130 (range 80–201) minutes and the median postoperative hospital stay 7 (range 5–95) days. In two patients, a postoperative complication occurred: one early stapler line leakage requiring laparotomy for re-suturing and one late stapler line leakage which led to a prolonged hospital stay and finally resulted in Billroth II gastrectomy. Both patients were obese (BMI > 30) and in both cases the tumour was located on the posterior stomach wall. In the former case, the leakage occurred on postoperative day 1 when the patient presented with severe abdominal pain and preseptic conditions. Diagnosis was made by application of methylene blue through the nasogastric tube and its detection through the indicative drainage which was still in place. The patient was immediately re-operated through a small laparotomy and the further clinical course was uneventful except for subcutaneous wound infection. The latter patient was first treated with re-laparoscopy on postoperative day 6 for removal and drainage of an intraabdominal abscess. On postoperative day 10, leakage was detected endoscopically and treated with endoluminal stenting. After gradual clinical improvement the patient was discharged on day 61 but was re-admitted few days later with peritonitic signs. Releakage was diagnosed and open subtotal gastrectomy had to be performed. Finally, the patient was discharged in good clinical condition 95 days after initial surgery.

Table 3 shows the results of the follow-up. After a median period of 18 (range 1–53) months, in none of the patients recurrence or distant metastases had been detected and all patients were alive. In patients with an intermediate risk GIST, median follow-up was 18 (range 6–46) months.

## 4. Discussion

The main principle of curative surgery for GISTs is en bloc resection with negative tumour margins and strict avoidance of intraoperative tumour rupture. Due to the extremely low frequency of lymphatic metastasis, lymphadenectomy is not required. Thus, segmental or wedge resection is the treatment of choice for tumours whose size and location technically allow for it [10, 11]. The aims of the present analysis were to assess if the mentioned surgical principles could be sufficiently met with a laparoscopic approach and to evaluate perioperative and oncological outcomes of patients resected with this method.

In line with previously published case series [12–28], our results support the application of laparoscopy for wedge resection of gastric GISTs. In all but one case deemed eligible for the procedure based on staging exams, the intervention could be performed without conversion to laparotomy. Resection with tumour-free resection margins was possible in all cases and there were no instances of intraoperative tumour rupture. Although there are no respective empirical data, we consider the usage of a retrieval bag for the removal of the surgical specimen essential in order to avoid spillage of tumour cells into the abdominal cavity or port sites, thus preventing metastasis. Even small GIST of 2-3 cm in size harbour the risk of malignant behaviour and consequently tumour resection should be performed according to standards of laparoscopic resection for GI malignancies.

In our series, overall perioperative morbidity was low with virtually no blood loss and satisfying operation times. As in other laparoscopic procedures, the existence of a “learning curve” must be assumed and it can be expected that operation times further decrease with growing experience [29–31]. The median length of hospital stay of our patients was relatively short, too. It can be speculated that patients could have been discharged even earlier and that the relatively lengthy stay was attributed to the rather recent introduction of the methodology at our centre.

In two patients, severe postoperative morbidity occurred due to leakage from the stapler line. In both cases, the tumour was partially located at the posterior wall of the stomach, and both patients were obese (BMI > 30). In such patients, laparoscopic resection is particularly challenging because it requires extended mobilisation of the stomach and preparation through the omental bursa, hampered by impaired visibility of the operation field. Technical difficulties in stapling and/or suturing might be the consequence. Both leakages led us to the routine use of fleece patches to strengthen the suture line and we strongly recommend their application in patients with the characteristics described. An alternative for the resection of lesions of the posterior gastric wall, especially for lesions located close to the gastro-esophageal junction, could be a transgastric approach through anterior gastrotomy [15, 20, 32–34]. This technique, however, is technically even more demanding [20], and there are reports of postoperative complications [32] and incomplete tumour resections [35]. As a complementary method, a combined endoscopic-laparoscopic approach can be used. We have applied this technique in several cases to facilitate tumour identification and lifting into the stapler predominantly in relatively small and non-protruding lesions. Its utility for resection of gastric GIST, especially for tumours in the proximal posterior part of the stomach, has been reported in several series [28, 36, 37]. Recently, a new technique of endoscopic full-thickness resection using a flexible stapler was described. This approach seems particularly useful in tumours of the posterior distal part of the stomach [38].

In summary, we do not consider any tumour location as a strict contraindication towards laparoscopy if the necessary experience is given and if the required precautions are met. Several additional technical approaches can be used to facilitate safe resection. Nevertheless, it is important to emphasize that the threshold for laparotomy should be rather low if intraoperative difficulties are encountered.

One concern about wedge resection of the stomach is the occurrence of postoperative gastric stenosis, especially when parts of the gastro-oesophageal junction or the pylorus are resected [17, 20, 23]. In our patients, we did not find any early postoperative stenosis. There were, however, only six cases where the tumour was located in the antrum and none with a tumour directly at the gastro-oesophageal junction. With regard to long-term functional results, we were not confronted with symptoms or endoscopical signs of reflux or stenosis during postoperative follow-up.

For tumours with a larger diameter and/or unfavourable location, primary wedge resection is often not possible and total or subtotal gastrectomy would be required for resection with tumour-free margins. For these cases, the NCCN guidelines [7] and ESMO recommendations [8] suggest neoadjuvant imatinib therapy to decrease tumour size, thus allowing for organ-preserving surgery. The feasibility and outcomes of this approach are currently evaluated in several clinical trials [39]. Our case series includes one patient who received six months of neoadjuvant imatinib treatment in the framework of the C STI571 BDE 43 trial, (“Apollon study”) [11]. Laparoscopic resection of an originally large GIST of the anterior corpus was made possible in this case thanks to a considerable shrinkage of the tumour.

In our series, 4 out of 21 tumours preoperatively suspected to be a GIST were histologically diagnosed as pancreatic heterotopia, gastric lipoma, schwannoma, and leiomyoma. Retrospectively, in these cases surgical resection would have not been required for oncological reasons. Preoperative histological diagnosis of submucosal gastric lesions is however not always feasible through endoscopic biopsy. Moreover, biopsy seems to be associated with a certain risk of tumour haemorrhage and dissemination [1]. Modern imaging techniques such as endosonography or CT gastrography with 3D reconstruction [40] can aid in making the preoperative diagnosis without being invasive towards the tumour. Nevertheless, a clear preoperative diagnosis will still not be possible in all cases of submucosal gastric tumours. We think that it is warranted to perform surgical excision of submucosal gastric lesions without prior histological ascertainment even if in a small percentage of cases the tumour is not a GIST. In fact, the latest ESMO recommendations [8] explicitly consider surgical excision of tumours without prior histological confirmation of GIST if they are larger than 2 cm or show an increase in size. Current NCCN guidelines even regard preoperative biopsy as not appropriate in easily resectable lesions and state that it is mandatory only if neoadjuvant treatment is planned [7]. Three of the four benign lesions were at a size of greater than 2 cm and by this met the criteria specified in both guidelines.

Follow-up did not show any local recurrence or distant metastases, and all patients were alive at the end of the follow-up period. This finding is similar to results from previous studies, which yielded excellent oncological long-term outcomes of laparoscopic resection of gastric GISTs [12, 14–16, 18–25, 32]. Median follow-up in our study was 18 months and some patients with low-risk GIST were not followed up through standardised exams (CT and endoscopy) but only indirectly by means of phone calls, which might not be sufficient to detect all tumour recurrences. Therefore, our results have to be interpreted with caution and no definite conclusions on the oncological safety of laparoscopic resection of gastric GIST can be made at this point. Even though in our series there were no recurrences in patients with intermediate and high risk tumours, we advocate that all patients with resected GISTs of these risk categories are included in clinical trials assessing the effect of adjuvant treatment [13, 39].

## 5. Conclusions

In summary, our findings support the application of laparoscopy for the resection of localised very low, low, and intermediate risk GISTs of the stomach. Special care needs to be employed when resecting lesions of the posterior gastric wall, which seem to be more prone to postoperative morbidity. The threshold to laparotomy should be rather low in case of intraoperative difficulties, especially in obese patients if there is no special experience in bariatric surgery. For large lesions and tumours in unfavourable locations such as the gastroesophageal junction or small curvature, neoadjuvant imatinib treatment might be an option to facilitate organ-preserving surgery.

To allow for a definitive recommendation of laparoscopic surgery as the new “gold standard” in the treatment of localised GISTs of the stomach, it would be highly desirable to have results from one or several randomised controlled trials [41]. The establishment of such trials, however, is not easily possible. Surgical procedures which have shown good results in nonrandomised studies and with which clinicians and patients have had an excellent (subjective) experience are often established as clinical standard without randomised controlled trials being conducted [42]. In our opinion, even if based only on data from retrospective analysis, laparoscopic wedge resection should be recommended as treatment of choice for localised GISTs of the stomach if the named limitations and precautions are borne in mind.

## Figures and Tables

**Table 1:** Classification of aggressive behaviour of GISTs proposed by Fletcher et al. [4].

	Tumour size (largest diameter)	Mitotic count per 50 high power fields
Very low risk	<2 cm	<5

Low risk	2–5 cm	<5

Intermediate risk	<5 cm	6–10
	5–10 cm	<5

High risk	>10 cm	any number
	any size	>10
	>5 cm	>5

**Table 2:** Perioperative and tumour characteristics of the patients.

Patient	Sex	Age	Tumour localisation	Histology	Max. tumour diameter (cm)	Mitotic figures/50 HPF	Risk classification^§^	Duration of surgery (minutes)	Postop. hospital stay	Postoperative morbidity
1	m	45	fundus	GIST	5	<5	low	165	5	none
2	m	79	fundus	GIST	4	2	low	94	7	none
3*	f	43	anterior corpus	GIST	0.8	<5	very low	89	5	none
4	f	56	greater curvature/ant. corpus	GIST	1.7	5	very low	175	6	none
5	f	55	lesser curvature	GIST	5.4	2	intermediate	113	6	none
6	m	59	posterior antrum	GIST	3.5	3	low	161	95	late stapler line leakage resulting in B-II-gastrectomy
7	f	74	lesser curvature	GIST	2.1	<5	low	130	7	none
8	m	45	anterior antrum	GIST	2.5	1	low	173	6	none
9	f	72	greater curvature/post. corpus	GIST	5.1	<5	intermediate	333	12	early stapler line leakage resulting in resuturing through laparotomy
10	f	52	anterior corpus	GIST	2	<2	low	125	6	none
11	f	46	greater curvature/ant. corpus	GIST	2.1	3	low	80	7	none
12^*#*^	f	66	antrum	GIST	2.9	2	low	112	9	none
13	m	82	lesser curvature	GIST	10	<5	intermediate	185	14	none
14	m	62	posterior corpus	GIST	2	<2	very low	201	8	none
15	m	64	anterior antrum	GIST	6	<5	intermediate	105	6	none
16*	f	67	lesser curvature	GIST	1.8	<5	very low	184	10	none
17^$^	m	66	anterior corpus	GIST	4	n/a	n/a	143	8	none

median	n/a	56	n/a	n/a	2.9	n/a	n/a	130	7	n/a

n/a: not applicable. *Additional cholecystectomy for cholecystolithiasis. ^*#*^Additional liver cyst deroofing. ^$^Due to preoperative imatinib treatment classification of aggressive behaviour not possible. ^§^see Table 1.

**Table 3:** Results of the follow-up of operated patients.

Patient	Sex	Age	Classification of aggressive behaviour^§^	Follow-up (months)	Tumour recurrence, metastases or death at end of follow-up?
1	m	45	low	44	no
2	m	79	low	40	no
3	f	43	very low	18	no
4	f	56	very low	27	no
5	f	55	intermediate	23	no
6	m	59	low	14	no
7	f	74	low	9	no
8	m	45	low	6	no
9	f	72	intermediate	6	no
10	f	52	low	53	no
11	f	46	low	10	no
12	f	66	low	12	no
13	m	82	intermediate	46	no
14	m	62	very low	47	no
15	m	64	intermediate	18	no
16	f	67	very low	1	no
17^$^	m	66	intermediate/high	5	no

median	n/a	59	n/a	18	n/a

n/a: not applicable; ^§^see Table 1; ^$^received preoperative imatinib treatment. Aggressive behaviour classified based on pre-treatment staging.
